# Prognostic analysis of esophageal cancer in elderly patients: metastatic lymph node ratio versus 2010 AJCC classification by lymph nodes

**DOI:** 10.1186/1477-7819-11-162

**Published:** 2013-07-18

**Authors:** Ji-Feng Feng, Ying Huang, Lu Chen, Qiang Zhao

**Affiliations:** 1 Department of Thoracic Surgery, Zhejiang Cancer Hospital, No.38 Guangji Road, Banshan Bridge, Hangzhou 310022, China; 2 Key Laboratory Diagnosis and Treatment Technology on Thoracic Oncology, Hangzhou, Zhejiang Province 310022, China; 3 Department of Nursing, Zhejiang Cancer Hospital, Hangzhou, China; 4 Department of Gynecologic Surgery, Zhejiang Cancer Hospital, Hangzhou, China

**Keywords:** Esophageal cancer, Lymph node ratio, Overall survival, TNM classification

## Abstract

**Background:**

Recent studies have proposed a new prognostic factor (metastatic lymph node ratio, or MLNR) for patients with esophageal cancer (EC). However, to the best of our knowledge, there have been no studies conducted to date regarding MLNR in elderly patients. The aim of this study was to determine the prognostic value of MLNR staging compared with the 2010 American Joint Committee on Cancer (AJCC) N staging in elderly patients with EC.

**Methods:**

From January 2001 to December 2009, a retrospective analysis of 132 consecutive patients older than 70 years of age with esophageal squamous cell carcinoma (ESCC) was conducted. Prognostic factors for disease-specific survival were analyzed. Receiver operating characteristic curves were also plotted to verify the accuracy of MLNR staging and N staging for survival prediction.

**Results:**

The disease-specific survival rates of N0, N1, N2 and N3 patients according to the AJCC Cancer Staging Manual Seventh Edition N staging were 65.5%, 42.9%, 22.2% and 0, respectively (N0 vs N1, *P* = 0.017; N1 vsN2, = 0.050; N2 vs N3, *P* < 0.001). The disease-specific survival rates of MLNR0, MLNR1, MLNR2 and MLNR3 patients were 65.5%, 45.0%, 21.1% and 0, respectively (MLNR0 vsMLNR1, *P* = 0.026; MLNR1 vs MLNR2, *P* = 0.033; MLNR2 vs MLNR3, *P* = 0.015). The areas under the curve were 0.731 for the 2010 AJCC N staging and 0.737 for the MLNR staging.

**Conclusion:**

MLNR is an independent predictor of survival in elderly patients with ESCC. MLNR staging predicts survival after EC similarly to the 2010 AJCC N classifications and should be considered an alternative to current N staging.

## Background

Esophageal cancer (EC) is the eighth most common type of cancer worldwide. In China, EC is the fourth most common cause of mortality, with 11 deaths per 100,000 in 2005 [1]. However, esophageal squamous cell carcinoma (ESCC) accounts for most EC cases in China, in contrast to the predominance of adenocarcinoma in the Western world [2]. Although advances have occurred in multidisciplinary treatment, surgical resection remains the treatment modality of choice. One of the main determinants of survival for patients undergoing esophagectomy is nodal status. However, the best method of characterizing the extent of local lymph node metastases remains an area of controversy.

Recent studies have proposed a new prognostic factor (metastatic lymph node ratio, or MLNR) for EC patients [3-5]. Controversy exists concerning the optimal cutoff points for the MLNR to predict overall survival. The different study sizes, variable inclusion criteria and, most important, unreliable statistical methods used to determine the cutoff points between groups have contributed to this controversy. However, to the best of our knowledge, there have been no studies conducted to date regarding MLNR in elderly patients with EC.

The aim of this study was to determine the prognostic value of MLNR staging compared with the 2010 American Joint Committee on Cancer (AJCC) classification system by lymph nodes in elderly patients with ESCC.

## Methods

### Patients

We conducted a retrospective analysis of patients treated from January 2001 to December 2009. The sample population comprised 132 patients older than 70 years of age with ESCC who underwent curative esophagectomy in the Department of Thoracic Surgery, Zhejiang Cancer Hospital, Hangzhou, China. Patients who had received pre- and or postoperative chemotherapy and/or radiotherapy were excluded. We also excluded patients with non-ESCC and gastroesophageal junction carcinoma, as well as patients who underwent surgical exploration without curative esophagectomy.

All of the above patients were followed up by posting letters or by telephone interviews. The last follow-up was on 30 November 2011. The clinicopathological and follow-up findings were collected and recorded in the database. All subjects gave their written informed consent to the study protocol, which was approved by the ethical committees of Zhejiang Cancer Hospital, Hangzhou, China.

### Surgery

All patients were treated with radical resection. The standard surgical approach consisted of a limited thoracotomy on the right side and intrathoracic gastric reconstruction (the Ivor Lewis procedure) for lesions at the middle or lower third of the esophagus. Upper-third lesions were treated by cervical anastomosis (the McKeown procedure). In our institution, two types of lymphadenectomy were carried out as a standard procedure for ESCC. The majority of patients underwent two-field lymphadenectomy. In this cohort of patients, thoracoabdominal lymphadenectomy was performed, including the subcarinal, paraesophageal, pulmonary ligament, diaphragmatic and paracardial lymph nodes, as well as those located along the lesser gastric curvature, the origin of the left gastric artery, the celiac trunk, the common hepatic artery and the splenic artery. Three-field lymphadenectomy was performed only if the cervical lymph nodes were thought to be abnormal upon preoperative evaluation. All of the patients included in the study were restaged according to the classification system of the seventh edition of the *American Joint Committee on Cancer (AJCC) Cancer Staging Manual* (AJCC Seventh Edition) [6].

### Statistical analysis

Statistical evaluation was conducted using SPSS version 17.0 software (SPSS Inc, Chicago, IL, USA). MLNR was defined as the ratio metastatic lymph nodes to total lymph nodes, and it was categorized into four groups (0,>0, ≤0.1, >0.1, ≤0.3 and >0.3) as described in a previous study [4]. As this series described the prognosis of elderly patients with ESCC, a disease-specific survival analysis would more appropriately indicate the impact of the N classification system on cancer-related prognosis. The disease-specific survival was calculated by using the Kaplan–Meier method, and the difference was assessed by using the logrank test. Univariate and multivariate analyses of Cox regression proportional hazards model were performed to evaluate the prognostic parameters for survival. Spearman’s correlation coefficient was calculated to assess the correlation related to MLNR. Receiver operating characteristic (ROC) curves were also plotted to verify the accuracy of MLNR staging and 2010 AJCC N staging for survival prediction. *P* < 0.05 was considered to be statistically significant.

## Results

### Patient characteristics

**Figure 1:**
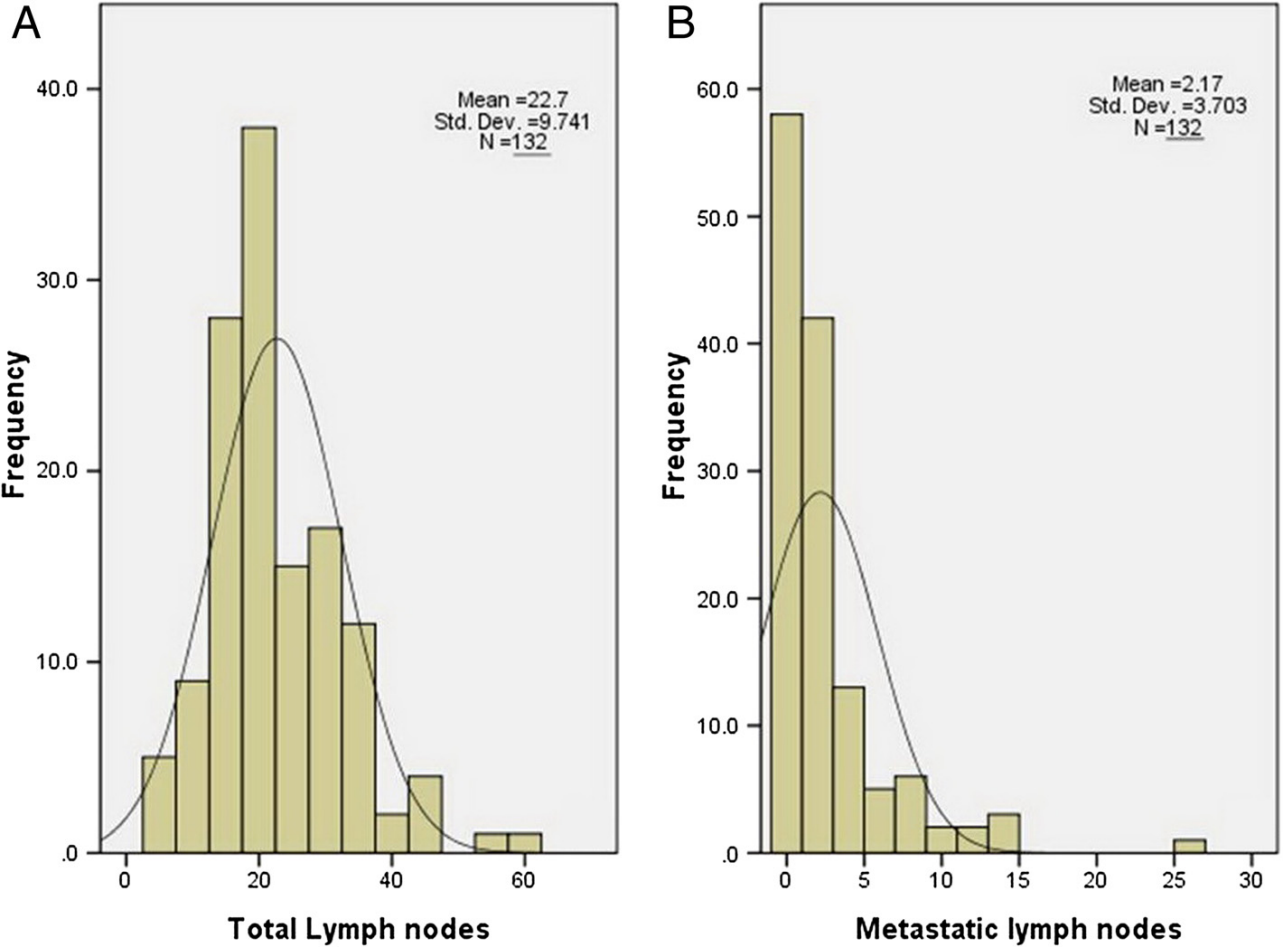
Histograms of the total (A) and metastatic (B) lymph nodes among surgery cohorts in ESCC patients.

The baseline characteristics are shown in Table 1. Among the 132 patients, 11 (8.3%) were women and 121 (91.7%) were men. Their mean age was 73.7 ± 2.6 years, with an age range of 70 to 85 years. The most common tumor locations were the middle and lower esophagus (95.5%). Total lymph node (TLN) harvest was highly variable (Figure 1A). A mean of 22.7 ± 9.7 nodes per patient was found during pathologic review (range: 6 to 61 nodes). The number of metastatic lymph nodes (NMLNs) per case ranged from 0 to 26 nodes (mean: 2.2 ± 3.7 nodes) (Figure 1B). The mean MLNR was 0.10 (range: 0 to 0.80). Of the 132 patients, 58 (43.9%) were classified as MLNR0, 40 (30.3%) as MLNR1, 19 (14.4%) as MLNR2 and 15 (11.4%) as MLNR3.

**Table 1:** **Baseline characteristics of 132 patients with ESCC**^a^

**Characteristics**	**Cases (n, %)**
Age (mean ± SD, yr)	73.6 ± 2.6
Gender	
Female	11 (8.3)
Male	121 (91.7)
Tumor size (mean ± SD, cm)	4.6 ± 1.7
Tumor location	
Upper	6 (4.5)
Middle	55 (41.7)
Lower	71 (53.8)
Histologic grade	
Well	17 (12.9)
Moderately	81 (61.4)
Poorly	34 (25.7)
Tumor grade	
T1	19 (14.4)
T2	16 (12.1)
T3	89 (67.4)
T4	8 (6.1)
N stage	
N0	58 (43.9)
N1	42 (31.8)
N2	18 (13.7)
N3	14 (10.6)
TLN (mean ± SD, *n*)	22.7 ± 9.7
NMLN (mean ± SD, *n*)	2.2 ± 3.7
MLNR	
MLNR0 (0)	58 (43.9)
MLNR1 (>0, ≤0.1)	40 (30.3)
MLNR2 (>0.1, ≤0.3)	19 (14.4)
MLNR3 (>0.3)	15 (11.4)

^a^*ESCC*, esophageal squamous cell carcinoma; *MLNR* metastatic lymph node ratio; *NMLN*, number of metastatic lymph nodes; *TLN*, total lymph node.

### Prognostic factors

Univariate analyses were performed to assess the predictive capability of each variable (Table 2). As expected, vessel involvement (*P* = 0.029), perineural invasion (*P* = 0.007), tumor grade (= 0.003), N stage (*P* < 0.001) and MLNR stage (*P* < 0.001) were predictive of survival. There were no significant differences in terms of age, gender, tumor size or tumor location. Significant factors were extracted for further analysis, which was conducted by using the multivariate Cox proportional hazards model to adjust for the effects of covariates (Table 3). In that model, we demonstrated that tumor grade (*P* = 0.003), N stage (*P* < 0.001) and MLNR stage (*P* < 0.001) were independent prognostic factors. However, better discrimination was found for the AJCC Nstage than MLNR classification in terms of hazard ratio (HR) (N2 vs N3).

**Table 2:** **Univariate Cox regression analysis of disease-specific survival**^a^

**Characteristics**	**Cases (n, %)**	**DSS (%)**	**HR (95% CI)**	***P *value**
Age (yr)				0.838
≤75	97 (73.5)	46.4	1.000	
>75	35 (26.5)	42.9	1.055 (0.629 to 1.770)	
Gender				
Female	11 (8.3)	54.5	1.000	0.474
Male	121 (91.7)	44.6	1.394 (0.561 to 3.460)	
Tumor size (cm)				0.081
≤5	91 (68.9)	52.7	1.000	
>5	41 (31.1)	29.3	1.524 (0.950 to 2.443)	
Tumor location				0.778
Upper	6 (4.5)	50.0	1.000	
Middle	55 (41.7)	45.5	0.938 (0.286 to 3.081)	0.916
Lower	71 (53.8)	45.1	1.114 (0.344 to 3.613)	0.857
Histologic grade				0.073
Well	17 (12.9)	58.8	1.000	
Moderately	81 (61.4)	48.1	1.587 (0.712 to 3.538)	0.259
Poorly	34 (25.7)	32.4	2.461 (1.053 to 5.752)	0.038
Vessel involvement				0.029
No	100 (75.8)	48.0	1.000	
Yes	32 (24.2)	37.5	1.786 (1.061 to 3.007)	
Perineural invasion				0.007
No	113 (85.6)	49.6	1.000	
Yes	19 (14.4)	21.1	2.198 (1.240 to 3.894)	
Tumor grade				0.003
T1	19 (14.4)	89.5		
T2	16 (12.1)	68.8	4.319 (0.854 to 12.727)	0.069
T3	89 (67.4)	33.7	7.731 (2.868 to 14.844)	0.003
T4	8 (6.1)	25.0	10.551 (2.969 to 21.902)	0.001
TLN (nodes)				0.729
≤18	47 (35.6)	44.7	1.000	
>18	85 (64.4)	45.9	0.905 (0.597 to 1.427)	
N stage				<0.001
N0	58 (43.9)	65.5	1.000	
N1	42 (31.8)	42.9	2.059 (1.136 to 3.732)	0.017
N2	18 (13.7)	22.2	4.122 (2.047 to 8.299)	<0.001
N3	14 (10.6)	0	19.108 (8.503 to 42.939)	<0.001
MLNR stage				<0.001
MLNR0	58 (43.9)	65.5		
MLNR1	40 (30.3)	45.0	1.963 (1.070 to 3.602)	0.026
MLNR2	19 (14.4)	21.1	4.142 (2.902 to 8.202)	<0.001
MLNR3	15 (11.4)	0	12.037 (5.866 to 24.698)	<0.001

^a^*CI*, confidence interval; *DSS*, disease-specific survival; *HR*, hazard ratio.

**Table 3:** **Multivariate Cox regression analysis of disease-specific survival**^a^

**Characteristics**	**HR (95% CI)**	***P *value**
Vessel involvement		0.667
No	1.000	
Yes	0.868 (0.456 to 1.652)	
Perineural invasion		0.205
No	1.000	
Yes	1.555 (0.786 to 3.075)	
Tumor grade		0.003
T1	1.000	
T2	2.177 (0.474 to 6.841)	0.198
T3	4.190 (0.733 to 10.148)	0.090
T4	7.170 (1.909 to 15.503)	0.010
N stage		<0.001
N0	1.000	
N1	1.315 (0.709 to 2.440)	0.385
N2	2.366 (1.143 to 4.897)	0.020
N3	16.474 (6.390 to 42.468)	<0.001
MLNR stage		<0.001
MLNR0	1.000	
MLNR1	1.173 (0.625 to 2.203)	0.619
MLNR2	2.678 (1.320 to 5.433)	0.006
MLNR3	7.860 (3.695 to 16.718)	<0.001

^a^*CI*, confidence interval; *HR*, hazard ratio.

### Disease-specific survival

**Figure 2:**
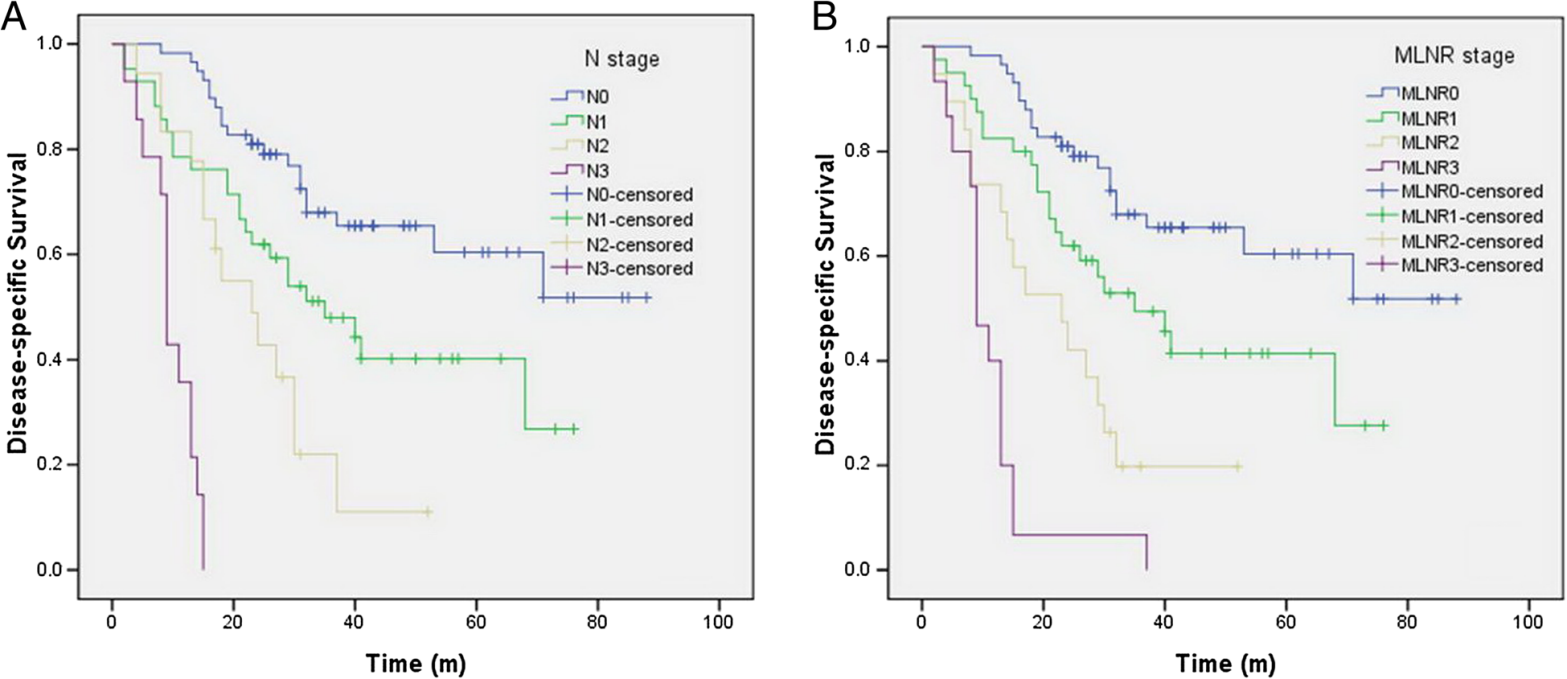
**Impact of AJCC Seventh Edition N staging (A) and MLNR staging (B) on disease-specific survival of ESCC patients who underwent radical resection. (A)** The disease-specific survival rates of N0, N1, N2 and N3 patients in AJCC Seventh Edition N staging were 65.5%, 42.9%, 22.2% and 0, respectively (N0 vs N1, *P* = 0.017; N1 vs N2, *P* = 0.050; N2 vs N3, *P* < 0.001). **(B)** The disease-specific survival rates of MLNR0, MLNR1, MLNR2 and MLNR3 patients were 65.5%, 45.0%, 21.1% and 0, respectively (MLNR0 vs. MLNR1, *P* = 0.026; MLNR1 vs MLNR2, *P* = 0.033; MLNR2 vs. MLNR3, *P* = 0.015).

The disease-specific survival rate was 45.5%. The survival curves developed according to the AJCC Seventh Edition N staging system, and the MLNR staging data are shown in Figure 2. The disease-specific survival rates of N0, N1, N2 and N3 patients according to AJCC Seventh Edition N staging were 65.5%, 42.9%, 22.2% and 0, respectively (N0 vs N1, *P* = 0.017; N1 vs N2, *P* = 0.050; N2 vs N3, *P* < 0.001). The disease-specific survival rates of MLNR0, MLNR1, MLNR2, and MLNR3 patients were 65.5%, 45.0%, 21.1% and 0, respectively (MLNR0 vs MLNR1, *P* = 0.026; MLNR1 vs MLNR2, *P* = 0.033; MLNR2 vs MLNR3, *P* = 0.015). The survival rates were similar between patients with N0 and N1 and those with MLNR0 and MLNR1, but the survival rates differed significantly between N1 vs N2 and MLNR1 vsMLNR2 (*P* = 0.050 vs. *P* = 0.033).

### Correlation related to MLNR

**Figure 3:**
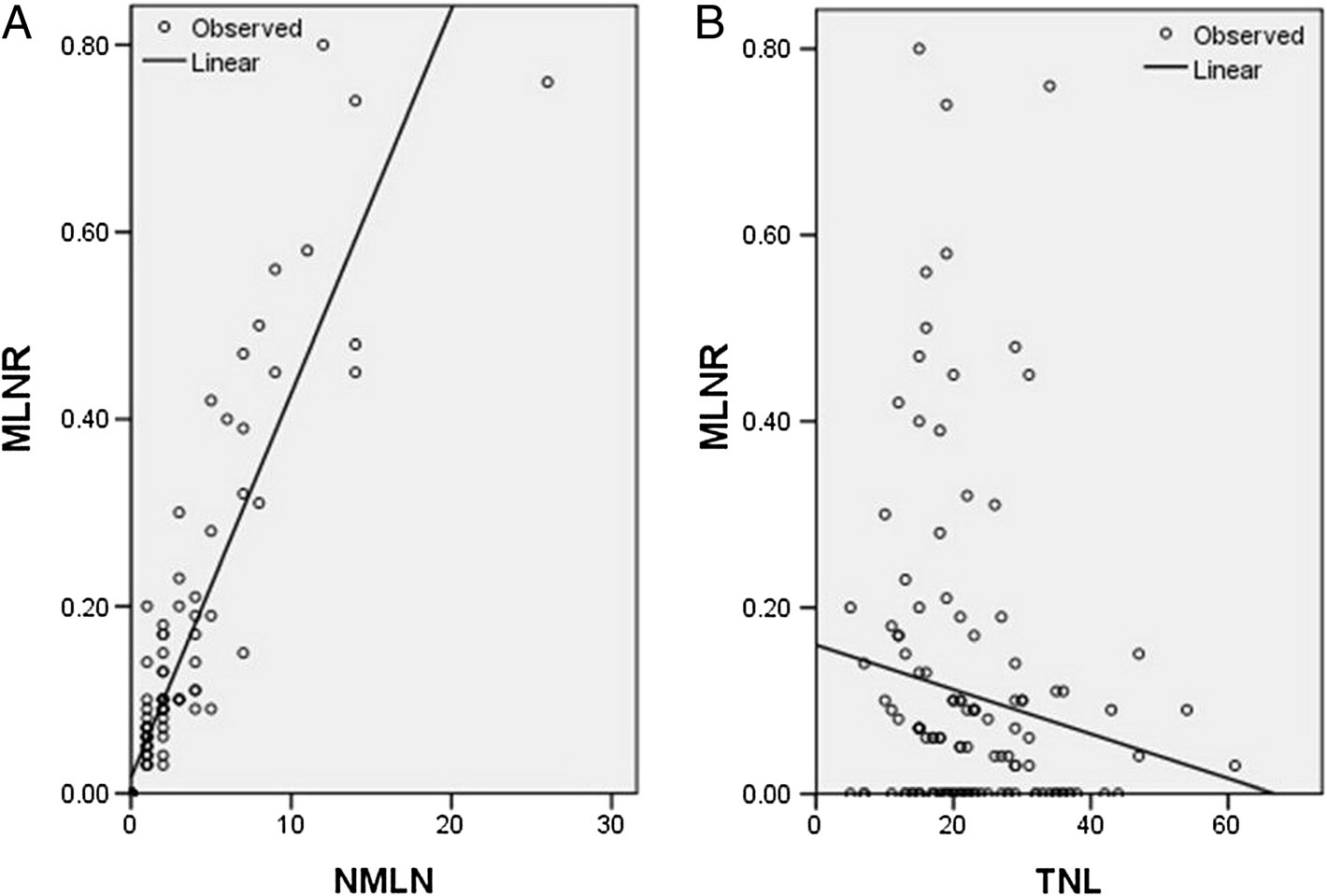
**Correlation related to MLNR. (A)** There was a positive correlation between the MLNR and NMLN (*r* =0.914, *P* < 0.001). **(B)** A negative correlation between MLNR and TLN, however, was not significant (*r* = −0.140, *P* = 0.110).

As expected, there was a positive correlation between the MLNR and NMLN (*r* = 0.914, *P* < 0.001) (Figure 3A). A negative correlation between MLNR and TLN, however, was not significant (*r* = −0.140, *P* = 0.110) (Figure 3B).

### ROC curve for disease-specific survival prediction

**Figure 4:**
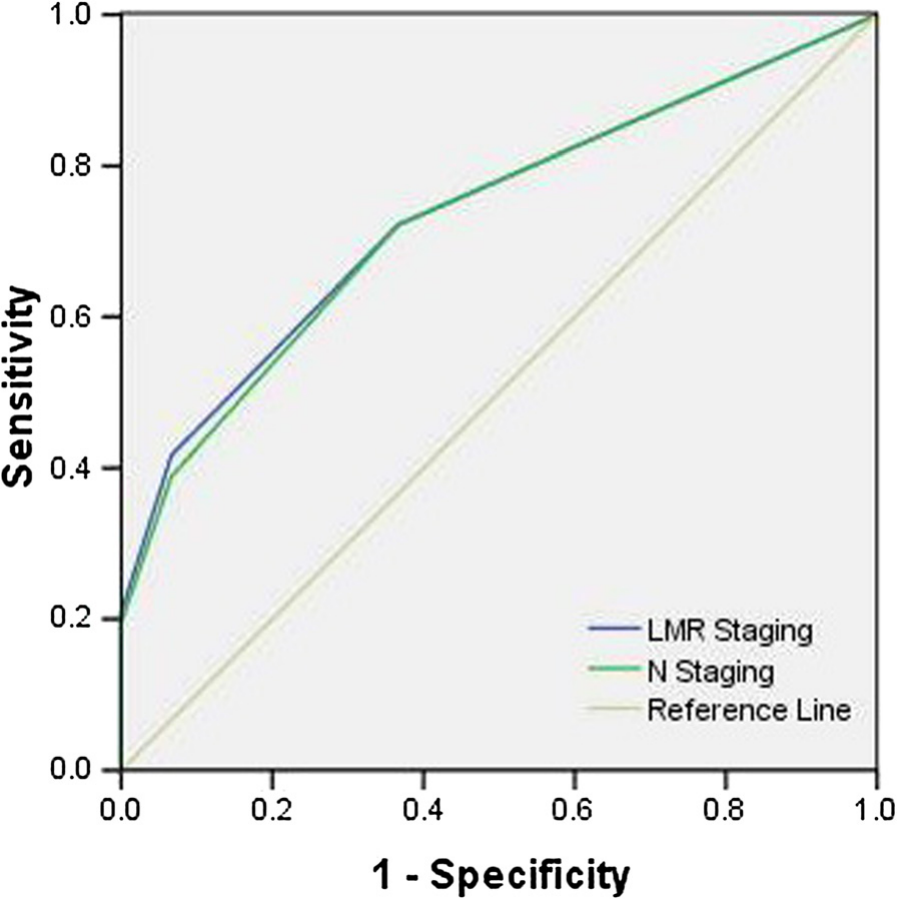
**ROC curve for survival prediction. **The ROC for MLNR staging is represented by the blue line with an AUC = 73.7%, and the ROC for AJCC Seventh Edition N staging is represented by the green line with an AUC = 73.1%.

The area under the curve (AUC) ratios were 0.731 (95%CI: 0.647 to 0.816, *P* < 0.001) for AJCC Seventh Edition N staging and 0.737 (95% CI: 0.653 to 0.821, *P* < 0.001) for the MLNR staging, indicating that the MLNR staging was similar to the AJCC Seventh Edition N staging system and could be used as an alternative prognostic staging tool for ESCC patients (Figure 4).

## Discussion

The aging of the population and a longer life expectancy have led to more elderly patients with cancers being referred for treatment. For many of them, in particular for those with EC, surgery remains the mainstay of treatment. There is no established cutoff to define a patient as “elderly” in relation to surgery, but most studies available to date have set the age limit at 70 years [7,8]. In our study, we determined the prognostic value of MLNR compared with N staging in EC patients older than 70 years of age. Our results suggest that MLNR might be a valuable prognostic factor of survival in elderly patients in EC. We conclude that MLNR staging predicts survival after EC similar to the AJCC Seventh Edition N staging classifications and should be considered as an alternative to current pathological N staging. To the best of our knowledge, our present studymay be the first to evaluate the value of MLNR vsAJCC Seventh EditionN staging in elderly patients with EC.

The ratio of metastatic to total lymph nodes (that is, the MLNR) has been shown to be a prognostic factor in EC, but the value of MLNR that is most predictive of survival is being debated. Most of the studies published to date concerning the MLNR and survival have been based on the AJCC Sixth Edition classification system [3-5]. Furthermore, in terms of MLNR and survival, some studies have classified the MLNR into three groups, whereas other studies have used two classifications. In our study, the MLNR was categorized by deciles into 0 (MLNR0), >0 to <0.1 (MLNR1), 0.1 to <0.3 (MLNR2) and ≥0.3 (MLNR3), based on the AJCC Seventh Edition classification system. We developed the MLNR intervals on the basis of our data to provide clinically relevant MLNR strata while probing to identify the subset of MLNR with the greatest predictive potential. In our study, the disease-specific survival rates of MLNR0, MLNR1, MLNR2 and MLNR3 patients were 65.5%, 45.0%, 21.1% and 0, respectively (MLNR0vs MLNR1, *P* = 0.026; MLNR1 vs MLNR2, *P* = 0.033; MLNR2 vs MLNR3, *P* = 0.015). Wilson *et al*. [9] classified 144 patients into 4 groups according to MLNR: 0, ≤25%, >25 to ≤50% and > 50%. Althoughan increasing MLNR was linearly associated with a worsening 5-yr survival rate in their study, statistical significance was not achieved (*P* = 0.153). Bogoevski *et al*. [10] also classified 235 patients into four categories according to MLNR: 0, <11%, 11% to 33% and >33%, which is similar to our findings.

The question of how many lymph nodes should be dissected has been a point of debate in previous studies. Rizk *et al*. [11] reported that the prognosis of patients after esophagectomy worsens significantly after four or more lymph nodes have metastases, irrespective of T stage. Greenstein *et al*. [12] and Yang *et al*. [13] recommended 18 nodes as the minimum number of resectable lymph nodes, whereas Peyre *et al*. [14] recommended a minimum of 23 regional lymph nodes. Attendees at a consensus conference of experts in 1995 suggested that accurate pathological staging of EC requires resection of at least 15 nodes [15]. The International Union Against Cancer (UICC) and AJCC have proposed that at least sixlymph nodes should be removed during resection of EC. Hu *et al*. [16] used a cutoff of six removed lymph nodesas the definition of adequate nodal dissection. Their results showed that patients with six or more lymph nodes dissected had a higher rate of positive lymph nodes identified (46.9% vs 30.3%) and an improvement in overall survival that was statistically significant in pathologically node-negative patients. Accordingly, we excluded patients who had fewer than sixdissected lymph nodes (range: 6 to 61). In our study, we did not find any survival rate difference when using a cutoff of 18 nodes (42.6% vs 44.7%, *P* = 0.741).

In the present study, the correlation related to MLMR was determined. As expected, we found that there was a positive correlation between MLNR and NMLN (*r* = 0.914, *P* < 0.001). There was a negative correlation between MLNR and TLN; however, the correlationwas not significant (*r* = −0.140, *P* = 0.110). ROC curves were plotted to verify the accuracy of MLNR staging and N staging for survival prediction. The AUCs were 0.731 for the 2010 AJCC N staging and 0.737 for the MLNR staging, indicating that the MLNR staging was similar to the AJCC Seventh Edition N staging system and could be used as an alternative prognostic staging tool for EC patients.

The potential limitations of the present study include the relatively small number of patients, the use of a retrospective analysis and the short duration of the mean follow-up. In addition, because the study used data from a single institution but with different pathologists and different surgeons, there may have been a lack of uniformity in measurement methods. Furthermore, owing to the limited number of patients in EC, our analysis may contain type I or type II errors. The results of the study should therefore be regarded with caution. Further studies are needed to explore its long-term effect.

## Conclusion

In summary, our study suggests that the survival rate of elderly patients with ESCC can be categorized into four groups: MLNR0 (0), MLNR1 (>0 to ≤0.1), MLNR2 (>0.1 to ≤0.3) and PLNR3 (>0.3). We conclude that MLNR is an independent predictor of survival in elderly patients undergoing esophagectomy for EC. MLNR staging predicts survival after EC similarly to the 2010 AJCC N classifications and should be considered as an alternative to current pathological N staging.

## Competing interests

The authors declare that they have no competing interests.

## Authors’ contributions

JFF conceived this study, collected data, performed analysis and drafted the manuscript. YH participated in the study design, literature search and study coordination. YH and LC performed data analysis and helped to draft the manuscript. QZ participated in the study design and helped to draft the manuscript. All authors read and approved the final manuscript.
